# Cognitive risk during underwater exposure: from environmental stressors to assessment and context-aware monitoring

**DOI:** 10.3389/fphys.2026.1888182

**Published:** 2026-08-07

**Authors:** Houyu Zhao, Longfei Wang, Yucheng Zhao, Nan Zhao, Shi Zhang, Yan Wang, Xuhua Yu, Yiqun Fang

**Affiliations:** 1 Department of Diving Medicine, Navy Special Medical Center, Naval Medical University, Shanghai, China; 2 Department of Underwater and Hyperbaric Medicine, Navy Special Medical Center, Naval Medical University, Shanghai, China; 3 Department of Medical Research, Naval Medical Specialty Center, Naval Medical University, Shanghai, China

**Keywords:** cognitive impairment, context-aware monitoring, critical flicker fusion frequency, diving physiology, EEG, heart rate variability, underwater exposure

## Abstract

Underwater exposure imposes a distinctive cognitive burden because divers must maintain performance while environmental and physiological safety margins can change rapidly. Safe performance depends on attention, psychomotor speed, executive control, working memory, spatial orientation, metacognition, and decision-making, each of which may be influenced by pressure, breathing gases, respiratory load, immersion, thermal stress, fatigue, and individual susceptibility. Evidence from recreational, technical, breath-hold, and saturation diving remains difficult to synthesize because studies differ in exposure mode, gas composition, timing, task selection, ecological validity, and operational endpoints. This narrative state-of-the-art review addresses three questions: which stressors shape cognitive risk, how risk is currently assessed, and how selected signals might contribute to future context-aware estimation without adding unsafe task burden. We describe the literature-identification strategy, define cognitive risk as a probabilistic and baseline-referenced state rather than a single test abnormality, and map vulnerable domains to operational hazards such as delayed response, navigation error, unsafe gas decisions, missed alarms, and delayed recovery. We critically evaluate behavioral tasks, CFFF, EEG/ERP, HRV, electrodermal activity, NIRS, respiratory CO_2_ monitoring, and wearable platforms, emphasizing feasibility, signal quality, nonspecificity, synchronization, individualized baselines, and validation against operational outcomes. Finally, we propose a staged pathway from dry chamber validation to wet-chamber or planned-pause testing and then to occupational or saturation settings. Selected physiological and behavioral signals may contribute to cognitive-risk estimation only after transparent interpretation and prospective validation against meaningful endpoints.

## 1 Introduction

Underwater operations require cognition, physiology, and environmental control to remain coordinated under rapidly changing conditions. A diver must navigate, communicate, monitor life-support equipment, regulate buoyancy, interpret environmental cues, and maintain contingency plans while exposed to pressure, altered breathing gases, immersion, thermal stress, limited visibility, restricted movement, and limited escape options (Clark, 2015; Piispanen et al., 2021; Sharma et al., 2023). For recreational, military, occupational, technical, and saturation divers, even a small delay in attention, gas management, or emergency decision-making can be amplified by depth, decompression obligation, equipment complexity, respiratory burden, and team dependence (Hobbs et al., 2014; Freiberger et al., 2016; Dunworth et al., 2017; Dreyer et al., 2024).

Underwater cognition therefore differs from generic laboratory cognitive performance. Attention, working memory, inhibition, processing speed, situation awareness, workload, physiological state, and decision-making operate as a closed-loop system (Endsley, 1995; Wickens, 2008). Attention supports monitoring of depth, time, gas supply, buddy position, task progress, and unexpected change. Executive control suppresses unsafe responses under anxiety, overload, or narcosis, a function that appears vulnerable during underwater exposure (Karakaya et al., 2021). Working memory supports navigation, decompression planning, and procedural recall, whereas metacognitive accuracy is essential because divers may not reliably recognize their own impairment during inert gas narcosis (Germonpré et al., 2017). Cognitive decline underwater should therefore be viewed not only as a lower test score, but as reduced reliability, self-detection, and resilience of task-relevant control (Ergen et al., 2017; Lafère et al., 2019).

The evidence base has expanded. Recent studies and reviews have described cognitive function in scuba, technical, and saturation diving, including alertness, memory, decision-making, executive function, event-related potentials, and functional connectivity (Germonpré et al., 2017; Steinberg and Doppelmayr, 2017; Karakaya et al., 2021; Sharma et al., 2023; Vrijdag et al., 2022a). Extreme exposures remain difficult to study, but a 45 ATA simulated saturation dive reported impaired numerical Stroop performance even in expert divers (Kageyama and Sawamura, 2024). Taken together, these findings suggest that underwater cognitive vulnerability is measurable and exposure dependent, although not uniform across domains or paradigms.

The field remains fragmented. Studies differ in wet versus dry exposure, at-depth versus post-dive testing, breathing gas, exercise state, thermal condition, expertise, and cognitive endpoint. Monitoring approaches are equally diverse: CFFF is used as a low-burden index of central arousal (Lafère et al., 2019; Mankowska et al., 2021), EEG/ERP can capture neurofunctional changes (Karakaya et al., 2021; Vrijdag et al., 2022a), and HRV has been explored in relation to operational performance. No single tool currently provides a validated, continuously deployable, and operationally robust measure of underwater cognitive risk.

**Figure 1:**
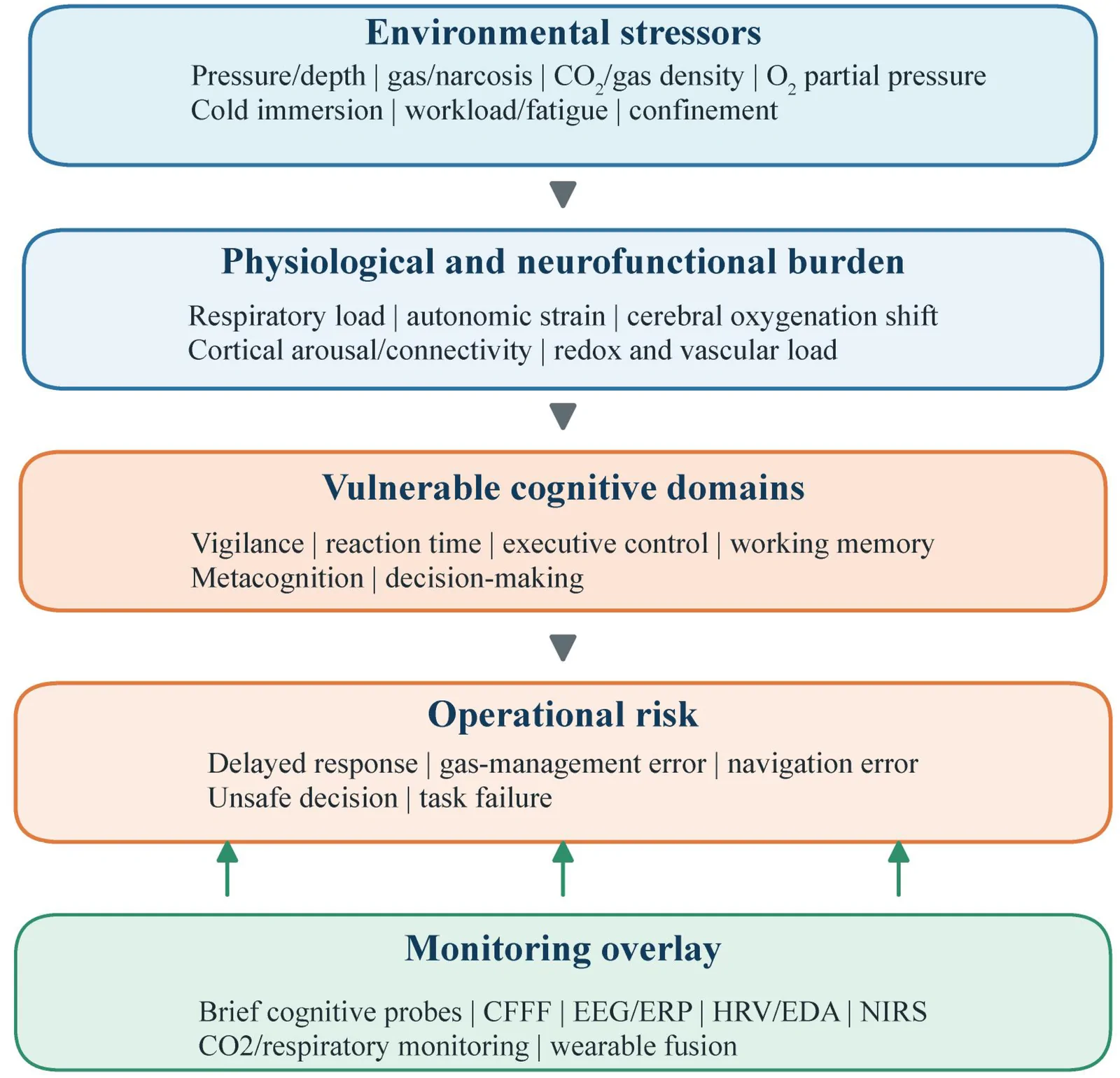
Conceptual risk cascade linking underwater environmental stressors to cognitive performance and operational risk. Environmental stressors may increase physiological and neurofunctional burden, which can affect vulnerable cognitive domains and safety-relevant performance. The arrows indicate a simplified pathway; feedback may occur among cognition, behavior, anxiety, breathing pattern, respiratory load, task demand, mitigation decisions, and environmental exposure.

This review has both a review component and a perspective component. The review component summarizes cognitive domains, environmental determinants, and assessment tools. The perspective component proposes a conceptual monitoring framework that should be read as a research roadmap rather than operational guidance. We distinguish established observations, emerging hypotheses, and future research recommendations throughout. The overall conceptual logic is summarized in **Figure 1**.

## 2 Scope and approach of this review

This narrative, state-of-the-art review is intended for diving physiology, diving medicine, human factors, neuroergonomics, and extreme-environment research. It provides a structured interpretive synthesis and research roadmap rather than a systematic evidence grade or practice guideline. A systematic review or meta-analysis was not attempted because exposure mode, depth, gas composition, exercise state, timing, participant expertise, and cognitive outcomes vary widely. Instead, the review is organized around a translational question: how can underwater cognitive risk be understood and studied in ways that may eventually improve diving safety and operational performance?

PubMed/MEDLINE, Web of Science, Scopus, and Google Scholar were searched from database inception to May 2026. Search terms combined exposure terms (diving, scuba, technical diving, saturation diving, freediving, underwater exposure, hyperbaric chamber, immersion), stressor terms (nitrogen narcosis, hypercapnia, carbon dioxide, oxygen partial pressure, hyperoxia, gas density, cold exposure, workload, fatigue), cognitive terms (cognition, cognitive impairment, vigilance, reaction time, executive function, working memory, metacognition, decision-making), and monitoring terms (CFFF, EEG, ERP, HRV, EDA, NIRS, respiratory monitoring, wearable sensor, passive BCI, neuroergonomics). Reference lists of key reviews and mechanistic papers were also screened. Selection followed an iterative narrative-review process rather than blinded duplicate screening; no formal excluded-study log was generated. Priority was given to human diving or hyperbaric studies with clearly described exposure conditions, objective cognitive or operational outcomes, time-resolved or at-depth measurements, and monitoring relevance. Studies were de-emphasized when exposure was poorly characterized, the outcome was remote from diving safety, or the monitoring implication was not interpretable.

Because this is not a systematic review, no pooled estimate or formal risk-of-bias score was calculated. This narrative approach has important limitations: study selection was not blinded, contradictory findings could not be resolved statistically, and conclusions should be read as a structured synthesis rather than a definitive evidence hierarchy. Contradictory findings were interpreted by comparing exposure characterization, timing of assessment, cognitive-domain specificity, ecological validity, and linkage to operational outcomes rather than by vote counting. We also applied a practical hierarchy of interpretation: respiratory CO_2_ and breathing load were treated as high-priority monitoring targets because they have direct physiological and safety relevance; behavioral tasks provide objective but task-specific evidence; CFFF, EEG/ERP, HRV/EDA, and NIRS are adjunct signals that require context and endpoint validation; biological markers are mainly mechanistic or recovery-context evidence. Cognitive performance refers to observed task output, cognitive impairment to an observed decline on a defined task, cognitive readiness to pre-dive preparedness, and cognitive risk to the probability that a diver’s current environmental, physiological, and behavioral state will degrade task-relevant cognition before an overt error occurs. Thus, cognitive risk is a probabilistic latent operational state, an engineering prediction variable, and a safety metric rather than a clinical diagnosis. Operationally, it should be estimated from individualized baseline deviation, exposure load, respiratory burden, autonomic or neurofunctional strain, task phase, subjective state, and recovery trajectory: cognitive risk = f(baseline deviation, exposure load, respiratory burden, autonomic/neurofunctional strain, task phase, subjective state, prior recovery). Thresholds should be validated against operational outcomes, not assumed from universal cutoffs. **Table 1** summarizes this evidence hierarchy and its interpretive limits.

**Table 1:** Evidence hierarchy and interpretive limits supporting the review framework.

Evidence cluster	Representative sources	Evidence strength in this review	Current inference	Main limitation	Interpretive use
CO_2_ and respiratory burden	Dunworth et al., 2017; Freiberger et al., 2016	Relatively direct physiological and safety relevance	CO_2_ and breathing load are high-priority variables	CO_2_, effort, anxiety, and workload often co-vary	Measure when feasible and validate against performance
Behavioral task evidence	Dalecki et al., 2012; Steinberg and Doppelmayr, 2017; Kageyama and Sawamura, 2024	Objective but task-specific	Selected domains can deteriorate during immersion or hyperbaric exposure	Tasks, timing, gas, and ecology are heterogeneous	Use as endpoint-specific probes, not global scores
Gas narcosis and metacognition	Hobbs et al., 2014; Germonpré et al., 2017; Vrijdag et al., 2022a	Moderate mechanistic and behavioral support	Narcosis may affect cognition and self-awareness	Wet/dry exposure and task differences limit generalization	Pair gas variables with domain-specific probes
CFFF	Balestra et al., 2012; Lafère et al., 2019; Mankowska et al., 2026	Adjunct arousal/neurofunctional evidence	Useful for low-burden state sampling	Not a global cognition measure; device and context dependent	Combine with tasks and exposure variables
EEG/ERP	Karakaya et al., 2021; Vrijdag et al., 2022a	Mechanistically informative in controlled settings	Can detect neurofunctional changes during hyperbaric exposure	Artifact, sensor, and ecological-validity constraints	Use primarily for chamber validation and selected field studies
HRV/EDA	Freiberger et al., 2024; Chen et al., 2025	Contextual autonomic evidence	May index strain trends related to workload or stress	Nonspecific to cognition and affected by breathing/cold/exercise	Interpret with respiratory, thermal, and task labels
NIRS and breath-hold monitoring	Eichhorn et al., 2015; McKnight et al., 2021	Useful in apnea and constrained contexts	Can track cerebral oxygenation trajectories	Sensor fixation, systemic physiology, and context specificity	Focus on hypoxia/apnea and recovery questions
Biological mechanisms	Vezzoli et al., 2024; Balestra et al., 2024; Zhao et al., 2024a, Zhao et al., 2024b	Mechanistic and recovery-context evidence	May help explain susceptibility or prolonged effects	Does not validate real-time cognitive-risk monitoring in humans	Use for post-dive recovery and mechanistic research

## 3 Cognitive domains affected by underwater exposure

Cognitive performance underwater cannot be reduced to a single score. Different stressors may affect different domains, and the same exposure can impair one process while sparing, or transiently improving, another. Apparent contradictions across studies may therefore reflect domain specificity, testing time, and task selection rather than true inconsistency.

Attention and vigilance are central because divers monitor multiple dynamic variables while filtering irrelevant stimuli. Reduced vigilance can delay detection of gas problems, depth deviations, equipment malfunction, or buddy distress. Cold exposure and shallow immersion can affect attention and processing speed even without deep pressure exposure, and nitrogen narcosis may add distractibility, overconfidence, and reduced situation awareness (Dalecki et al., 2012; Falla et al., 2021).

Reaction time and psychomotor performance matter because many underwater tasks are time constrained. Simple reaction time is useful for repeated low-burden testing, whereas discrimination reaction time, tracking, and multitasking better approximate operational performance. Comparisons of reaction-time measures and CFFF indicate that motor speed, visual processing, and arousal are related but separable constructs (Tikkinen et al., 2016).

Executive function, including inhibitory control, cognitive flexibility, conflict monitoring, and planning, is especially operationally relevant. Divers at 20 m water depth showed selective executive-function impairment (Steinberg and Doppelmayr, 2017), and numerical Stroop testing detected changes during a 45 ATA simulated saturation dive (Kageyama and Sawamura, 2024). These paradigms are attractive because they probe conflict processing with relatively simple stimulus-response demands.

Executive vulnerability is not limited to one task or exposure model. Response-selection paradigms suggest that nitrogen narcosis can alter sensorimotor decision stages rather than simply slow motor output (Meckler et al., 2014). Across open-water and chamber studies, the most sensitive endpoint varies with depth, gas, timing, and task complexity, arguing against a single global cognition score (Tikkinen et al., 2016; Brebeck et al., 2017; Germonpré et al., 2017; Lafère et al., 2019).

Working memory supports navigation, decompression planning, equipment sequencing, and procedural recall. It is commonly assessed with digit span, Corsi block-tapping, n-back, and spatial tasks. Findings are inconsistent, probably because cognitive load, timing, and practice effects differ. Monitoring should therefore match the probe to the operational task rather than treat working memory as a universal marker.

Decision-making and metacognition are critical because divers must recognize when their own performance is deteriorating. Gas narcosis is hazardous when subjective confidence fails to decline with objective performance. Studies suggest that objective and subjective measures may not fully align during air or nitrox exposures (Hobbs et al., 2014; Germonpré et al., 2017), making impairment-awareness mismatch a relevant monitoring target.

Longer-term and repeated-exposure findings are harder to interpret. Some studies link diving exposure to neuropsychological, neurofunctional, or white-matter outcomes, whereas others are weaker or confounded by training, selection, decompression history, vascular risk, and exposure heterogeneity (Slosman et al., 2004; Hemelryck et al., 2014; Bast-Pettersen et al., 2015; Coco et al., 2019; Rosén et al., 2022). These data should not be overread as proof of chronic injury in all divers, but they justify longitudinal and recovery-focused designs.

### 3.1 Mapping cognitive domains to operational risk

A domain-to-risk mapping is needed because operational incidents rarely arise from one isolated cognitive function. Gas-management errors may combine attention to gauges, working-memory updating, metacognitive recognition of narrowing awareness, and decision-making under social or mission pressure. Navigation deviation may depend on spatial working memory and divided attention, whereas delayed alarm response may depend on vigilance, psychomotor speed, respiratory strain, and thermal stress. Future protocols should predefine the domain expected to contribute to each operational outcome.

This mapping also clarifies validation. A simple reaction-time probe may detect vigilance loss or psychomotor slowing, but it cannot validate a model intended to prevent unsafe gas decisions. Conversely, a short Stroop or response-inhibition probe may represent conflict monitoring but may be less sensitive to hand cooling or display constraints. **Table 2** summarizes this mapping as a guide for future protocol design.

**Table 2:** Proposed mapping between cognitive domains and operational risk endpoints for future protocol design.

Cognitive domain	Operational risks	Candidate endpoint	Recommended probe or context
Vigilance/sustained attention	Missed alarm, delayed detection of gas or depth deviation, buddy-distress oversight	Missed signal rate; time to detect abnormal display or alarm	Brief vigilance probe; event detection during simulated monitoring
Reaction time/psychomotor speed	Delayed response to equipment, communication, or ascent/descent cues	Simple or choice reaction time; response delay to standardized cue	Low-burden repeated probe; separate motor and cognitive components when cold or gloves are present
Executive control	Unsafe impulse, poor inhibition under narcosis/anxiety, inappropriate emergency action	Stroop/interference cost; response-inhibition errors; rule violation	Short Stroop, Flanker, Simon, or go/no-go probe during chamber or planned pause
Working memory/spatial orientation	Navigation deviation, procedure omission, decompression-plan error	Route deviation; recall of gas/time plan; sequence error	Spatial span, n-back, route-memory, or procedure-recall probe matched to mission
Metacognition/decision-making	Failure to recognize impairment, overconfidence, unsafe gas or ascent decision	Mismatch between self-rating and objective performance; unsafe choice	Performance-confidence rating plus objective task and operational scenario

This mapping is intended to guide future protocols and requires validation before use in risk estimation or decision support.

## 4 Environmental determinants of cognitive risk

**Table 3** summarizes the major environmental stressors, candidate mechanisms, affected domains, and monitoring implications discussed in this section.

**Table 3:** Environmental stressors and cognitive implications during underwater exposure.

Stressor	Diving context	Cognitive domains affected	Candidate mechanisms	Representative evidence	Monitoring implications	Priority
Depth/pressure	Scuba, technical, saturation	Executive control, reaction time, psychomotor performance	Increased gas partial pressures, gas density, decompression obligation	Steinberg and Doppelmayr, 2017; Kageyama and Sawamura, 2024	Continuously label depth, pressure, phase, and decompression obligation	High
Nitrogen narcosis	Compressed-air and trimix diving	Judgment, memory, calculation, inhibitory control, metacognition	Inert gas effects on CNS function and network connectivity	Clark, 2015; Hobbs et al., 2014; Vrijdag et al., 2022a	Pair gas exposure with brief executive probes and EEG/CFFF where feasible	High
Gas composition	Air, nitrox, heliox, trimix	Error rate, arousal, cognitive-domain-specific effects	Narcotic potency, oxygen partial pressure, gas density	Germonpré et al., 2017; Lafère et al., 2019; Sharma et al., 2025	Report partial pressures and gas density; avoid gas-label-only interpretation	Medium-high
Hypercapnia/respiratory burden	Exercise at depth, rebreathers, high-density gas	Attention, planning, psychomotor performance, situational awareness	CO_2_ retention, increased work of breathing, dyspnea, anxiety	Dunworth et al., 2017; Freiberger et al., 2016	Prioritize CO_2_ and breathing-load monitoring when feasible	High
Oxygen partial pressure	Nitrox, HBO, rebreathers, saturation	Arousal, central excitability, task-specific performance	Hyperoxia, chemoreflex modulation, CNS oxygen toxicity risk	Vrijdag et al., 2022b; Möller et al., 2023	Interpret arousal metrics with oxygen exposure and CNS toxicity risk in view	Context dependent
Cold immersion	Open-water and polar/temperate diving	Attention, processing speed, executive function, dexterity-mediated performance	Thermal stress, discomfort, peripheral cooling, arousal changes	Falla et al., 2021; Piispanen et al., 2021	Combine thermal, manual-performance, workload, and cognitive measures	Context dependent
Workload and fatigue	Operational, military, rescue, prolonged operations	Vigilance, decision-making, multitasking, working memory	Exercise, sleep loss, cumulative stress, fatigue	Möller et al., 2021, Möller et al., 2023	Use phase labels, workload ratings, fatigue measures, and longitudinal baselines	High in prolonged work
Breath-hold hypoxia/hypercapnia	Freediving	Executive control, awareness, hypoxic blackout risk	Apnea, cerebral oxygenation changes, blood-gas shifts	McKnight et al., 2021; Paganini et al., 2026	Integrate NIRS/SpO_2_, apnea phase, symptoms, and recovery trajectory	High in freediving

### 4.1 Hyperbaric pressure and depth

Depth is the most visible determinant of underwater risk, but cognitive effects arise through several pathways: gas partial pressures, gas density, work of breathing, decompression obligation, thermal exchange, and equipment complexity. Depth is therefore useful operationally, but effects attributed to depth may actually reflect gas, respiratory load, or task context.

At recreational and technical depths, compressed-air diving increases nitrogen partial pressure and may induce inert gas narcosis. At extreme depths, saturation exposure adds confinement and cumulative physiological load. A simulated 440 m sea-water saturation dive at 45 ATA found impaired numerical Stroop performance in expert divers, suggesting that training does not eliminate hyperbaric cognitive vulnerability (Kageyama and Sawamura, 2024).

Technical diving adds complexity because gas choice, decompression obligation, equipment configuration, bailout planning, and task loading change simultaneously. Future studies should therefore report depth and pressure together with gas composition, inspired partial pressures, estimated gas density, exercise state, temperature, and task demands. Without these details, depth-related cognitive effects remain difficult to interpret.

### 4.2 Inert gas narcosis and breathing-gas composition

Nitrogen narcosis can affect reasoning, memory, calculation, judgment, mood, motor behavior, and self-awareness, with risk generally increasing with depth during compressed-air diving. Its operational hazard is not only performance decline, but also reduced recognition of decline while safety-critical decisions continue (Clark, 2015).

Neurophysiological studies provide mechanistically informative, but context-limited, evidence. Auditory ERP changes during simulated 500 kPa air exposure and EEG functional-connectivity changes during air breathing at 608 kPa suggest altered cognitive processing under hyperbaric nitrogen (Karakaya et al., 2021; Vrijdag et al., 2022a). These findings support EEG/ERP as research tools, but not yet as deployable operational monitors.

Breathing-gas composition may modify cognitive risk through narcotic potency, gas density, oxygen partial pressure, and respiratory mechanics. Air, nitrox, heliox, and trimix are not interchangeable exposures. Open-water and chamber studies indicate gas-specific effects, but differences in inspired partial pressures, task timing, and subjective state limit direct comparison (Brebeck et al., 2017; Germonpré et al., 2017; Sharma et al., 2025).

Future studies should report gas composition quantitatively rather than categorically. Inspired partial pressures, gas density, and breathing resistance are likely more informative than labels such as air, nitrox, or trimix, especially when comparing dry chambers with wet dives.

Nitrox illustrates this caution. It may reduce venous gas bubbles in selected simulated protocols (Souday et al., 2016), but decompression advantage should not be conflated with a validated cognitive benefit across all tasks and depths.

### 4.3 Hypercapnia, respiratory burden, and gas density

Carbon dioxide retention is a major determinant of underwater cognitive risk. Hypercapnia can arise from elevated inspired CO_2_, scrubber failure, dead space, inadequate ventilation, high gas density, breathing resistance, exercise, or behavioral suppression of breathing. It can escalate rapidly and may interact with anxiety, dyspnea, and oxygen-toxicity risk (Dunworth et al., 2017).

CO_2_ effects are not identical to nitrogen or oxygen effects. Hyperbaric studies indicate that N_2_, CO_2_, and O_2_ can affect attention, memory, planning, and psychomotor outcomes differently (Fothergill et al., 1991; Freiberger et al., 2016; Gill et al., 2014). CO_2_ should therefore be measured where feasible rather than inferred from symptoms alone.

Respiratory burden links physiology to cognition. Increased gas density and resistance can raise breathing effort, promote CO_2_ retention, increase anxiety, and reduce spare attentional capacity. Inspired or expired CO_2_, ventilation, respiratory rate, work of breathing, and gas density should be recorded whenever feasible, particularly in rebreather, technical, and high-workload dives.

A key research need is to separate hypercapnia from effort. Cognitive risk may reflect elevated CO_2_, high physical workload, or both. Combining respiratory monitoring with brief cognitive probes and autonomic signals could help identify when respiratory burden becomes cognitively consequential.

### 4.4 Oxygen partial pressure, hyperoxia, and central excitability

Oxygen is neither simply protective nor harmful. Inspired oxygen partial pressure varies with depth and breathing mixture, and its cognitive effects depend on level, duration, susceptibility, and interaction with nitrogen, CO_2_, and workload. Higher oxygen partial pressure may reduce inert gas fraction in some mixtures, but hyperoxia can also alter central excitability and increase oxygen-toxicity risk in susceptible contexts (Wingelaar et al., 2017; Ciarlone et al., 2019; Vrijdag et al., 2022b).

Oxygen-related neural effects may be misread as improved alertness, reduced narcosis, or emerging hyperexcitability depending on the outcome measured. CFFF studies under normobaric and hyperbaric oxygen suggest arousal changes, but not simple cognitive enhancement (Sharma et al., 2024).

High oxygen partial pressure should not be assumed to improve cognition uniformly. Oxygen exposure should be modeled as a state variable when interpreting CFFF, EEG, subjective alertness, or cognitive probes during oxygen-rich decompression, rebreather use, or hyperoxic exercise.

### 4.5 Cold immersion and thermal stress

Cold exposure can degrade cognition through discomfort, pain, altered arousal, motor impairment, distraction, and changes in skin and core temperature. It may impair attention, processing speed, executive function, and memory (Falla et al., 2021), while also reducing dexterity, display interaction, regulator comfort, and task persistence.

Cold is also an indirect cognitive stressor because it can increase perceived workload, breathing discomfort, and fatigue. Arctic and cold-water diving studies highlight the need to report temperature, exposure duration, suit type, hand cooling, and task type alongside cognitive outcomes (Piispanen et al., 2021).

Immersion itself is not neutral. Five-meter water immersion can impair overall cognitive performance, sustained attention, and tracking, and task complexity may influence the size of the deficit (Dalecki et al., 2012). These findings caution against attributing all effects to gas or pressure.

For monitoring, thermal and immersion variables should be integrated rather than treated as nuisance factors. A slowed response during cold-water tool use may reflect cognitive slowing, hand cooling, visibility, stress, or their combination.

### 4.6 Physical workload, fatigue, and prolonged underwater operations

Exercise has bidirectional effects on cognition. In general exercise science, acute exercise may modestly improve selected executive-function or reaction-time outcomes, but effects depend on intensity, task, timing, and training status (Chang et al., 2012; McMorris and Hale, 2012; Ludyga et al., 2016; Garrett et al., 2024). Underwater exercise is different because it can increase CO_2_ production, ventilation demand, breathing discomfort, perceived exertion, fatigue, and attentional narrowing, especially with immersion, equipment load, increased gas density, or breathing resistance (Freiberger et al., 2016; Dunworth et al., 2017; Pattyn et al., 2018). Studies of submersion and hyperoxic underwater exercise show that cognitive responses may differ from dry laboratory expectations (Möller et al., 2021, Möller et al., 2023).

Fatigue may be more important than acute task load in prolonged operations, but it is harder to measure. It includes physical fatigue, sleep restriction, cumulative stress, repeated decompression demands, and psychological strain. In saturation diving, confinement, altered sleep, noise, social stress, and long exposure windows may produce fluctuations not captured by single pre- and post-dive tests (Sharma et al., 2023; Kageyama and Sawamura, 2024). Broader fatigue research suggests that cognitive and physical fatigue interact through perceived exertion, self-regulation, and sustained attention (Marcora et al., 2009; Van Cutsem et al., 2017; Pattyn et al., 2018).

Workload assessment should be explicit. NASA-TLX can separate mental demand, physical demand, temporal demand, effort, performance, and frustration (Hart and Staveland, 1988; Hart, 2006). In diving, these dimensions may diverge: a chamber task can impose high mental demand with little movement, whereas current-exposed work can impose high physical and respiratory demand with secondary cognitive effects. Workload reports should be paired with objective markers such as exercise intensity, ventilation, respiratory rate, CO_2_, heart rate, HRV, task duration, sleep history, and environmental exposure.

### 4.7 Saturation diving and confined hyperbaric exposure

Saturation diving combines depth, duration, confinement, sleep disruption, decompression time, team dependence, and constrained working conditions. Simulated 45 ATA saturation exposure has been associated with impaired cognitive performance, while commercial-diver studies highlight the importance of subjective state, fatigue, and chamber environment (Imbert et al., 2018; Kageyama and Sawamura, 2024).

Saturation studies are limited by small samples, logistical barriers, and difficulty standardizing exposure. This supports the rationale for continuous low-burden sampling, but also means that chamber environmental records, sleep/activity monitoring, brief executive probes, HRV, respiratory indicators, and subjective fatigue reports require prospective validation before operational use.

### 4.8 Breath-hold diving and hypoxia-related risk

Breath-hold diving should be treated separately from scuba and saturation diving. Dominant stressors include apnea, hypoxia, hypercapnia, hydrostatic pressure, bradycardia, peripheral vasoconstriction, cerebral blood-flow redistribution, and sometimes decompression stress. Recent reviews emphasize rapid blood-gas changes, neurovascular responses, and persistent knowledge gaps (Tetzlaff et al., 2021; Paganini et al., 2026). NIRS work in elite freedivers illustrates feasibility of cerebral oxygenation monitoring under specialized conditions (McKnight et al., 2021).

Freediving is useful for monitoring development because hypoxia and hypercapnia evolve rapidly and can be measured alongside neurofunctional signals. Event-related EEG and NIRS studies show that prolonged breath-holding or deep breath-hold dives can modulate neurocognitive and cerebral-oxygenation markers in experienced divers (Eichhorn et al., 2015; Steinberg and Doppelmayr, 2019; McKnight et al., 2021).

The apnea literature also emphasizes individual reserve and adaptation. Spleen and lung volumes predict apnea performance (Schagatay et al., 2012), and freedivers may show distinctive cerebral metabolism, cerebrovascular reactivity, vascular adaptation, or antioxidant responses during repeated hypoxic-hypercapnic exposure (Sureda et al., 2015; Tanaka et al., 2016; Vestergaard and Larsson, 2019). These adaptations should not be generalized uncritically to scuba or saturation diving.

For this review, breath-hold diving provides both a model of extreme physiological compensation and a reminder that monitoring thresholds are context specific. Mechanisms from freediving should not be transferred directly to compressed-gas or saturation diving.

Swimming and aquatic-exercise research reinforces this systems view: immersion, exercise, breathing pattern, and aquatic posture can influence cerebrovascular and cognitive function even outside compressed-gas diving (Shoemaker et al., 2019).

## 5 Assessment methods for underwater-related cognitive risk

**Table 4** summarizes candidate cognitive assessment tools, their advantages, limitations, and feasible use contexts.

**Table 4:** Cognitive assessment tools used in diving and hyperbaric studies.

Tool/task	Target domain	Advantages	Limitations	Underwater feasibility	Best-use scenario/recommended use
Simple reaction time	Psychomotor speed	Brief, repeatable, easy to automate	Practice effects; motor contamination; low domain specificity	High	Repeated low-burden probe for trend detection
Discrimination/tracking tasks	Sensorimotor coordination, sustained attention	Closer to operational performance	Affected by dexterity, visibility, and equipment	Moderate	Controlled chamber or field protocols emphasizing operational performance
Stroop/Simon/Flanker	Inhibitory control and conflict processing	Sensitive to executive changes; feasible in chambers	Requires display-response reliability; practice effects	Moderate	Core executive probe for chamber, wet, or planned-pause testing
Digit span/Corsi/n-back	Verbal and spatial working memory	Domain-specific; compact	May be less sensitive than RT/executive measures in some exposures	Moderate	Navigation, planning, or procedure-heavy missions
MATB-II or micro-MATB	Multitasking and operational performance	Higher ecological validity	Longer, more complex, harder underwater	Low to moderate	Model training and validation in chambers or simulators
CFFF	Visual processing/cortical arousal proxy	Simple and portable	Confounded by illumination, method, age, ocular factors; not global cognition	Moderate	Adjunct arousal signal within multimodal assessment, not a global cognition proxy
Waterproof digital testing	Multiple domains	Potential for in-water assessment	Interface reliability, glove use, pressure/waterproofing	Emerging	Field feasibility studies after interface validation

### 5.1 Behavioral cognitive tasks

Behavioral tests remain the foundation of cognitive assessment because they are interpretable, inexpensive, and directly related to task performance. Reaction-time tasks are brief and repeatable; Stroop tasks assess conflict processing; digit span, Corsi, n-back, and spatial tasks assess working memory; and tracking or dual-task paradigms approximate motor-cognitive coordination. Tasks should be chosen according to the cognitive domain most relevant to the mission.

Behavioral tasks also have limitations. Practice effects, motor slowing, hand cooling, display visibility, glove use, buoyancy, and touchscreen reliability can contaminate interpretation. Post-dive testing may miss transient at-depth impairment or introduce recovery effects, whereas at-depth testing may interfere with safety. Protocols should therefore use brief, robust, domain-specific probes with repeated baselines and automated quality-control flags.

### 5.2 Stroop and executive-function paradigms

Stroop-type paradigms assess inhibitory control and conflict processing with simple stimuli. Their use at 20 m water depth and during simulated 45 ATA saturation exposure supports feasibility across different contexts (Steinberg and Doppelmayr, 2017; Kageyama and Sawamura, 2024). The challenge is to keep probes short enough for safety while retaining sensitivity to meaningful deterioration.

### 5.3 Multitasking and operational-performance batteries

Operational performance underwater is inherently multitask: divers track depth, time, gas, communication, navigation, body position, and task progress. Multitasking batteries therefore offer greater ecological validity than isolated tasks. MATB-II-like frameworks, which include tracking, monitoring, communication, and resource management, are especially relevant when physiological monitoring is linked to operational performance rather than isolated cognitive scores (Freiberger et al., 2024).

### 5.4 Critical flicker fusion frequency

CFFF is widely used in diving and hyperbaric research because it is simple, non-invasive, and repeatable. It is often interpreted as a marker of cortical arousal or central nervous system alertness (Mankowska et al., 2021). Studies using CFFF under different gas mixtures and after scuba diving explain why it remains attractive for field research (Balestra et al., 2012; Lafère et al., 2019).

Its interpretation should be restrained. CFFF is best treated as a complementary neurofunctional index rather than a substitute for cognitive testing. It enables repeated low-burden sampling of arousal state, but is affected by instructions, illumination, device characteristics, circadian state, learning, ocular factors, and threshold definition (Mankowska et al., 2021, Mankowska et al., 2026; Muth et al., 2026).

Recent work reinforces this caution. Oxygen exposure can alter CFFF responses across normobaric and hyperbaric conditions, and correlations between CFFF components and executive or memory measures appear variable (Sharma et al., 2024; Mankowska et al., 2025, Mankowska et al., 2026). Thus, CFFF meaning depends on gas, light, device, participant, and task context.

Accordingly, CFFF should be combined with exposure data, brief cognitive probes, and physiological signals rather than used as a stand-alone proxy for global cognition.

### 5.5 Underwater-compatible digital testing

Digital testing can bridge laboratory neuropsychology and operational monitoring. Waterproof tablets or pressure-tolerant interfaces could allow short cognitive probes during chamber exposures or planned underwater pauses. A recent case study identified feasibility but also practical limits in stimulus presentation and touch-response reliability (Yoshimura and Takahashi, 2025). Future systems need glove-compatible interfaces, high-contrast displays, low burden, timing calibration, and physiological-data integration.

### 5.6 Timing, baselines, and interpretation

Assessment timing strongly shapes interpretation. Pre-dive testing captures fatigue, sleep, anxiety, and baseline readiness; in-dive testing captures acute exposure effects but is constrained by safety; immediate post-dive testing may detect residual effects; and delayed testing may reflect recovery, fatigue, or decompression-related symptoms (Dreyer et al., 2024). Rigorous protocols should include repeated individualized baselines, exposure-phase labels, and explicit handling of learning effects.

## 6 Physiological and neurofunctional monitoring strategies

**Table 5** summarizes the main physiological and neurofunctional monitoring approaches, including their signals, limitations, and current status.

**Table 5:** Physiological and neurofunctional monitoring approaches.

Monitoring method	Signal type	Target risk	Strengths	Limitations	Current status
EEG	Neurofunctional connectivity, spectral dynamics, ERP components	Nitrogen narcosis, cortical arousal, stimulus processing	Direct brain-state information	Setup burden, artifacts, waterproofing, pressure tolerance	High mechanistic value; operational readiness remains early
ERP	P300/MMN-like indices of cognitive processing	Attention and stimulus evaluation	Objective and domain interpretable	Needs controlled stimuli; artifacts	Useful for validation; limited immediate in-dive readiness
HRV	Autonomic regulation	Stress, workload, fatigue, impaired performance risk	Wearable, continuous, low burden	Nonspecific; affected by breathing, cold, exercise	Useful for contextual trend monitoring within multimodal models
EDA	Sympathetic arousal	Stress and workload	Continuous, complementary to HRV	Water and motion challenges; nonspecific	Adjunct workload/stress signal; interpretation depends on context
NIRS/fNIRS	Cerebral oxygenation/hemodynamics	Hypoxia, cerebral stress, frontal activation	Useful in breath-hold and chamber contexts	Waterproofing, motion, hair, pressure effects	High for hypoxia/apnea questions; water deployment remains constrained
Respiratory CO_2_/ventilation	Inspired or expired CO_2_, breathing pattern	Hypercapnia and respiratory burden	Targets a known critical risk	Sensor integration and calibration underwater	High practical relevance when reliable sensing is available
Wearable platforms	Multisensor fusion	General cognitive-risk estimation	Scalable and longitudinal	Validation, data quality, interpretability	Research-stage; useful only after endpoint validation

### 6.1 EEG and event-related potentials

EEG is mechanistically valuable because it measures neurofunctional state directly, but most evidence remains chamber-based, proof-of-concept, or dependent on controlled stimuli. Functional connectivity at 608 kPa air exposure and auditory ERP changes during simulated hyperbaric nitrogen exposure suggest sensitivity to nitrogen narcosis and altered stimulus processing (Karakaya et al., 2021; Vrijdag et al., 2022a). These findings support EEG/ERP as research tools; they do not establish deployable in-dive monitoring or direct identification of cognitive impairment.

Field translation requires waterproofing, pressure tolerance, electrical safety, electrode stability, limited setup time, artifact control, ecological validity, and timely interpretability. A chamber signal is not automatically useful during a working dive with movement, bubbles, equipment noise, thermal stress, and task switching. EEG and ERP are therefore among the most informative mechanistic tools, but remain constrained for continuous operational use.

Signal-quality control should be a core requirement. In diving-relevant EEG/ERP, motion, ocular and facial-muscle artifacts, regulator or mask movement, electrode displacement, perspiration, pressure effects, water sealing, cable movement, and equipment noise can mimic or obscure neurocognitive effects. Wearable EEG improves portability but may reduce channel count, sampling flexibility, and artifact tolerance. Out-of-lab artifact-correction and wearable-EEG benchmarking work shows the need for online artifact management, device comparison, and neurometric reliability checks (Ronca et al., 2024, Ronca et al., 2026a).

EEG protocols should report electrode type, fixation, impedance or contact-quality criteria, sampling rate, filtering, artifact correction, retained data percentage, synchronization, and whether features were computed from artifact-free windows only. Any EEG-derived alarm should include signal-confidence flags and validation against prespecified task or operational endpoints.

### 6.2 HRV, electrodermal activity, and autonomic markers

Autonomic markers are attractive because they can be measured continuously and non-invasively. HRV reflects autonomic regulation, workload, stress, breathing patterns, and recovery state. Diving studies suggest associations between HRV features, operational performance, and physiological stress responses, but HRV should be used within multimodal models rather than as a stand-alone cognitive marker (Freiberger et al., 2024; Chen et al., 2025).

General HRV methodology supports caution because indices are strongly affected by respiration, posture, workload, stress, and recovery state (Laborde et al., 2017; Shaffer and Ginsberg, 2017). In underwater settings, these influences are part of the operational state. HRV is most useful when modeled with breathing pattern, CO_2_ risk, workload, temperature, and task performance.

EDA may complement HRV by indexing sympathetic arousal, but neither HRV nor EDA should be treated as a direct marker of cognitive impairment. Autonomic changes may reflect cognitive strain, emotion, cold, hypercapnia, exercise, pain, anxiety, breathing pattern, recovery, or equipment discomfort. Their value lies in synchronization with dive phase, respiratory variables, thermal exposure, workload, subjective state, and task performance (Ronca et al., 2026b).

### 6.3 NIRS and cerebral oxygenation

NIRS can monitor cerebral oxygenation and hemodynamics, especially in frontal regions relevant to executive control, but it does not directly measure cognition. It has been used during deep breath-hold diving in elite freedivers (McKnight et al., 2021), and may be useful in breath-hold, dry hyperbaric, or limited-movement chamber contexts. Barriers in scuba and occupational diving include sensor fixation, pressure tolerance, motion artifacts, water sealing, hair interference, thermal stress, equipment interference, and interpretation under changing oxygen and CO_2_ levels.

Interpretation requires care. fNIRS signals are affected by extracerebral blood flow, motion, optode placement, hair, and systemic physiology (Pinti et al., 2020). Diving amplifies these limitations because pressure, water sealing, facial equipment, exercise, and CO_2_ can all influence cerebral and peripheral hemodynamics.

NIRS should be viewed as a cerebral-state signal rather than a direct cognitive test. Reduced cerebral oxygenation may indicate hypoxic risk, but stable oxygenation does not guarantee preserved executive function. Like HRV, NIRS becomes useful when combined with exposure data and brief cognitive probes.

### 6.4 Respiratory CO_2_ monitoring and breathing-load assessment

Respiratory monitoring is one of the strongest candidates for future underwater cognitive-risk research because CO_2_ retention and breathing load have direct physiological and safety relevance. CO_2_ retention can arise from equipment failure, high work of breathing, exercise, or behavioral patterns and can rapidly degrade mental and physical performance (Dunworth et al., 2017). Inspired CO_2_, end-tidal CO_2_, ventilation, respiratory rate, gas density, and work of breathing would directly address a known safety threat, but sensor robustness and equipment integration remain major constraints.

Because subjective symptoms may not detect CO_2_-related cognitive risk early enough, respiratory metrics should be incorporated into future estimation models when feasible. A diver with rising CO_2_, increasing respiratory effort, deteriorating HRV, and slowed reaction time represents a different risk state from isolated mild slowing after repetitive testing, but this combined interpretation still requires prospective endpoint validation.

### 6.5 Wearable platforms and multimodal risk estimation

Wearable technology is expanding in diving, including devices for breath-hold applications, heart rate, and oxygen saturation (Vinetti et al., 2020; Bube et al., 2022; Beatty et al., 2024; Park et al., 2025). For cognitive risk, however, physiological signals must be validated against task-relevant cognitive and operational outcomes.

The next step is not simply to add sensors. A useful system must define what each signal means, when it should be interpreted as potentially relevant, and how false alarms will be controlled. Without such validation, wearable monitoring risks becoming data-rich but operationally weak.

**Figure 2:**
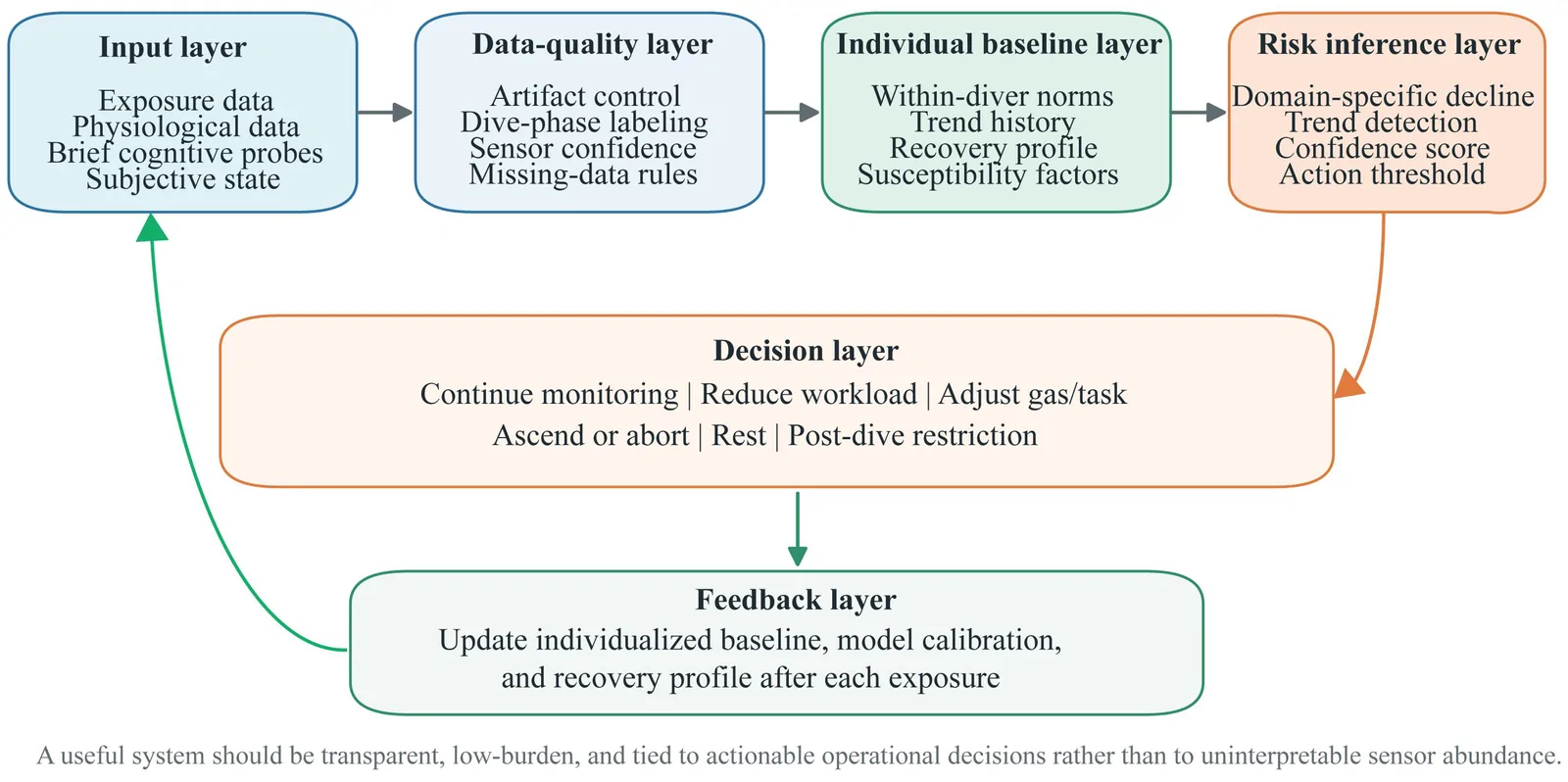
Proposed architecture for context-aware underwater cognitive-risk estimation. Exposure data, physiological signals, cognitive probes, and subjective state require artifact control, synchronization, baseline referencing, and feasibility checks before they can be translated into interpretable risk estimates. The architecture highlights key methodological requirements for future validation studies.

A proposed architecture could include environmental exposure data, physiological signals, and brief cognitive probes. Environmental data include depth, pressure, phase, gas, temperature, and workload proxies; physiological signals may include HRV, respiratory variables, EEG-derived metrics, NIRS, EDA, and oxygen saturation; cognitive probes should be short and domain specific. As outlined in **Figure 2**, these streams would require data-quality checks and individualized baselines before contributing to risk estimates. Such systems might eventually support continued monitoring, workload reduction, gas switch, ascent, rest, or post-dive recovery restriction, but only after prospective validation.

### 6.6 Feasibility staging for underwater monitoring

Feasibility differs substantially across settings. Dry hyperbaric chambers are suitable for mechanism testing and synchronized EEG/ERP or NIRS recording, but cannot reproduce movement, buoyancy, visibility, thermal, and equipment constraints. Wet chambers and planned underwater pauses can test waterproof interfaces, display-response reliability, and brief probes with lower safety risk. Continuous open-water or occupational monitoring is currently most realistic for passive exposure data, respiratory variables, heart rate or HRV, and selected low-burden wearable outputs; continuous EEG/ERP/NIRS remains research-stage unless signal quality and safety are demonstrated.

A method feasible during a decompression stop may be inappropriate during navigation, rescue, tool use, current exposure, or emergency ascent. Future studies should label each method as feasible now, chamber-only, feasible during planned underwater pauses, or speculative for continuous real-dive deployment. **Table 6** summarizes the corresponding feasibility and validation considerations.

**Table 6:** Feasibility and validation considerations for candidate monitoring components.

Component	Near-term feasible setting	Main constraints	Core validation endpoint
Exposure and equipment data	Continuous open-water, chamber, and saturation settings	Synchronization and standardized phase labels	Depth/phase-linked association with operational events
Brief cognitive probes	Dry chamber, wet chamber, planned underwater pauses	Practice effects, interface reliability, safety burden	Meaningful within-diver change; response delay; error rate
CFFF	Chamber and selected field protocols	Illumination, ocular factors, device thresholds	Arousal-state change as adjunct, not global cognition
HRV/EDA	Continuous passive monitoring	Nonspecificity: exercise, cold, CO_2_, anxiety, pain, recovery	Context-aware strain trends with respiratory and task labels
Respiratory CO_2_/breathing load	High priority when sensor integration is reliable	Sensor calibration, water sealing, dead space, equipment integration	Hypercapnia or breathing-load risk; relation to performance decline
EEG/ERP	Dry chamber; experimental wet settings; limited real-dive deployment	Waterproofing, pressure, artifacts, electrode stability, safety	Signal-quality-controlled association with attention/executive outcomes
NIRS/fNIRS	Freediving, chamber, and controlled underwater protocols	Fixation, hair, motion, systemic physiology, waterproofing	Cerebral oxygenation trajectory and recovery, especially apnea/hypoxia
Wearable fusion model	Research-stage; staged validation required	Data quality, synchronization, individual baselines, false alarms	Association with task failure, unsafe decision, navigation deviation, missed alarm, or delayed recovery

The matrix summarizes feasible testing settings, key constraints, and candidate validation endpoints that should be addressed before operational implementation.

### 6.7 Relation to passive BCI and neuroergonomics

The framework aligns with passive brain-computer interface and neuroergonomics research, which infers operator state from neurophysiological and physiological signals without deliberate commands. These fields have developed methods for monitoring workload, vigilance, stress, mental effort, and operator capacity in aviation, driving, surgery, and industry (Borghini et al., 2022; Bagheri and Power, 2022; Zhou et al., 2022). Diving shares the need for state estimation but adds pressure, immersion, respiratory constraints, water sealing, and limited interfaces. Underwater monitoring should borrow validation logic from these fields while avoiding direct transfer of thresholds from dry or seated tasks.

A useful underwater system should be transparent and operator centered. It should indicate whether risk is driven by respiratory burden, environmental exposure, autonomic strain, cognitive-probe deterioration, poor signal confidence, or their combination. Without interpretability, multimodal fusion may increase false alarms and distrust rather than improve safety.

## 7 Emerging biological mechanisms

This review is not primarily mechanistic, and biological mechanisms should be interpreted as context for susceptibility, recovery, and prolonged effects rather than as evidence for real-time cognitive-risk monitoring in human divers. Diving physiology involves pressure, gas kinetics, vascular responses, oxidative stress, decompression stress, and inflammatory responses (Bosco et al., 2018; Vezzoli et al., 2024). Dopamine/BDNF findings may offer a neurochemical bridge between narcosis-related physiology and cognition, although operational interpretation remains preliminary (Bosco et al., 2023).

The redox-vascular literature is relevant mainly to recovery, resilience, and repeated exposure. Diving can produce adverse stress responses or adaptive physiological responses depending on exposure profile, fitness, and recovery state (Perovic et al., 2014). Biomarkers should therefore be interpreted as context-dependent indicators of load and adaptation rather than simple evidence of injury.

Saturation and scuba studies have reported oxidative-stress biomarker changes across prolonged or depth-varying exposures (Mrakic-Sposta et al., 2020; Marchetti et al., 2022). These markers are not cognitive tests, but they may help explain recovery differences, prolonged fatigue, symptoms, or delayed readiness for complex tasks.

Field studies provide systemic context rather than direct cognitive-monitoring validation. Repeated or deep dives can be accompanied by endothelial dysfunction, oxidative stress, vascular gas emboli, and antioxidant responses, and integrated physiological-biochemical monitoring is feasible in real dive settings (Obad et al., 2010; Sureda et al., 2012; Marinovic et al., 2012; Balestra et al., 2024). These findings do not directly measure cognition, but they support including vascular and biological context when recovery or repeated exposure is a concern.

Vascular and neuroinflammatory pathways are emerging but should be interpreted cautiously. Decompression stress, endothelial activation, microbubbles, cerebral oxygenation changes, and blood-brain barrier vulnerability may contribute to post-dive symptoms or longer-term effects in susceptible contexts. Animal studies suggest pathways linking prolonged underwater stress, hippocampal vulnerability, microglial activation, and cognition, but should not be overgeneralized to human divers (Zhao et al., 2024a, Zhao et al., 2024b).

Immediate task performance may not fully capture biological burden, but current biological evidence does not justify real-time human cognitive-risk classification. Future studies should test whether physiological monitoring, biochemical markers, and cognitive recovery curves can improve post-dive risk assessment.

## 8 Practical implications and future directions

### 8.1 Pre-dive screening

Pre-dive assessment may need to move beyond general medical fitness in occupational or research contexts. Sleep, fatigue, stress, anxiety, medication, dehydration, prior dives, recent illness, and sensitivity to narcosis or CO_2_ can affect cognitive readiness. A short baseline battery covering reaction time, executive control, and subjective fatigue could contextualize in-dive or post-dive changes, but individualized thresholds require validation.

### 8.2 In-dive monitoring

In-dive monitoring must be low burden and should not distract from safety-critical tasks. The most realistic near-term approach is passive exposure and physiological monitoring, with brief cognitive probes only during planned pauses or chamber intervals. Systems should flag patterns that justify attention, workload reduction, or post-dive reassessment, not issue black-box operational commands.

### 8.3 Post-dive recovery assessment

Post-dive monitoring matters because impairment may persist after surfacing. Residual narcosis, vascular gas emboli, fatigue, and inflammatory or oxidative responses may affect recovery (Dreyer et al., 2024; Balestra et al., 2024). Post-dive tests should distinguish transient at-depth impairment, decompression-related symptoms, fatigue, and delayed recovery.

### 8.4 Standardization and validation

A standardized underwater cognitive-risk battery should include a small number of tasks covering vigilance, reaction time, executive control, and working memory, plus subjective fatigue and workload. The battery should be brief, repeatable, device-stable, and usable across dry chambers, wet chambers, and field conditions; it should support comparison rather than replace mission-specific assessment.

### 8.5 Toward individualized cognitive-risk estimation

Individualized cognitive-risk estimation is a future goal rather than a current operational capability. Divers differ in fitness, experience, anxiety, ventilatory response, narcosis susceptibility, cold tolerance, sleep, and learning effects. Machine-learning models may eventually integrate exposure, physiology, and performance, but require large, well-labeled datasets, transparent features, and validation against meaningful endpoints. Interpretability is essential; black-box scores without confidence or driver information should not guide safety-critical decisions.

### 8.6 Ideal study methodology

An ideal future study would be staged, prospective, and validation oriented. Stage 1 would establish repeated dry baselines across separate days to quantify intra-individual variability, practice effects, sleep/fatigue influences, and device reliability. Outputs would include stable baseline distributions for reaction time, executive control, vigilance, workload, HRV, respiratory variables, and any EEG/NIRS features.

Stage 2 would use controlled dry hyperbaric chamber exposures with standardized pressure, gas mixture, inspired partial pressures, CO_2_ or ventilation load, temperature, workload, and testing time. The goal would be feasibility, signal quality, domain sensitivity, and dose-response characterization, not operational deployment.

Stage 3 would test only components that passed stage-2 feasibility and signal-quality criteria in wet-chamber or open-water trials. Cognitive probes should be brief, prespecified, and limited to planned pauses. Waterproofing, display readability, response reliability, glove use, buoyancy, motion artifacts, and workload interference would become primary endpoints.

Stage 4 would evaluate occupational or saturation settings for longitudinal reliability, recovery curves, false-alarm burden, user acceptance, and decision usefulness. Core validation outcomes should include task failure, response delay, navigation deviation, gas-management error, unsafe ascent/descent behavior, missed alarm, expert-rated performance, abnormal recovery, and restriction from safety-sensitive work.

### 8.7 Research roadmap and priority areas

**Figure 3:**
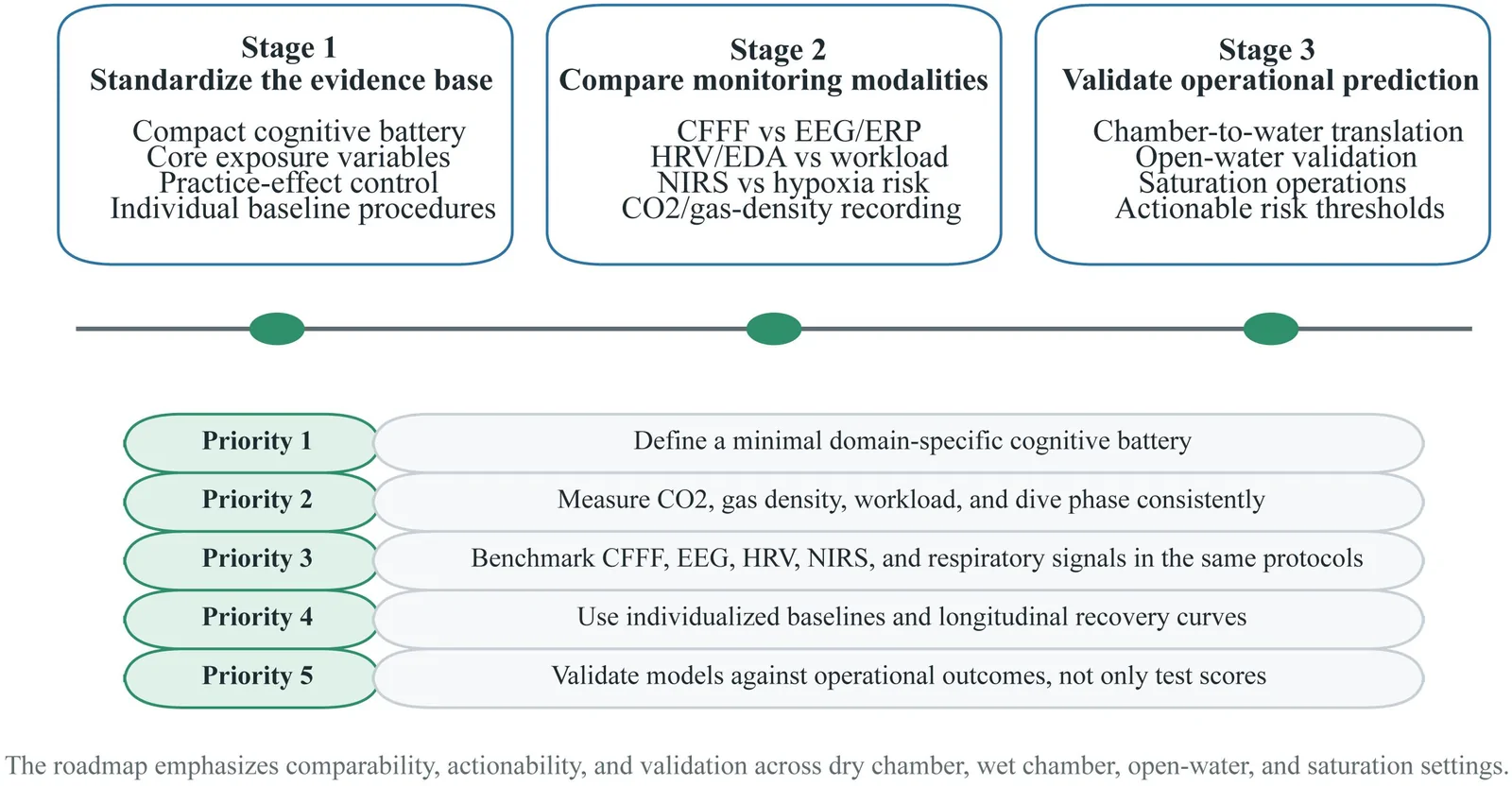
Research roadmap for underwater cognitive-risk assessment and monitoring. Future work should progress from standardized cognitive batteries and reporting variables, to direct comparison of monitoring modalities within the same protocols, and then to baseline-referenced estimation models evaluated across chamber, open-water, and saturation settings.

**Figure 3** translates the discussion into a staged roadmap. The field does not need more unrelated tests; it needs comparable protocols that connect exposure, physiology, cognition, and operational outcome. A minimal core dataset should include participant expertise, exposure profile, gas variables, CO_2_/respiratory burden, thermal exposure, workload, cognitive probes, physiological signal quality, and operational endpoints.

A staged strategy would reduce the gap between laboratory and field research. Chamber studies suit mechanism and signal validation; wet-chamber and open-water studies test usability and ecological validity; saturation or occupational studies are needed for longitudinal reliability and decision usefulness. Each stage should have explicit stop/go criteria before a tool is treated as operationally informative.

### 8.8 Research gaps and reporting standards

Several gaps remain urgent: real underwater studies are fewer than chamber studies; deep and saturation studies are necessarily small; CO_2_, workload, sleep, and thermal variables are often underreported; and few studies link signals to operational endpoints. Most importantly, multimodal signals have not yet been prospectively validated for safety-critical cognitive-risk classification. This limitation should guide interpretation of the proposed framework.

A practical reporting standard would improve comparability. Future studies should report participant expertise, pressure profile, wet/dry exposure, gas and inspired partial pressures, estimated gas density, CO_2_ and respiratory variables, temperature, workload, timing of cognitive tests, baseline procedures, device characteristics, signal-quality criteria, and operational endpoints. This would allow future systematic reviews or meta-analyses to distinguish robust findings from context-specific effects.

## 9 Conclusion

Underwater cognitive risk is a multidimensional physiological, neurofunctional, and operational problem shaped by pressure, gases, nitrogen narcosis, CO_2_ retention, oxygen partial pressure, immersion, cold, workload, fatigue, and individual susceptibility. Cognitive performance, cognitive impairment, cognitive readiness, and cognitive risk should be distinguished: impairment is observed task decline, whereas risk is a probabilistic, baseline-referenced state that may precede overt error.

The main contribution of this review is to organize current evidence and outline a staged research roadmap for underwater cognitive-risk assessment and monitoring. Future work should move from isolated pre- and post-dive tests toward integrated, context-aware, staged validation. Environmental exposure data, respiratory and autonomic measures, selected neurofunctional signals, and brief cognitive probes may become useful when quality controlled, synchronized, interpreted against individualized baselines, and evaluated against operational endpoints. **Figures 1**-**3** and **Tables 2**, **6** are intended to support protocol design and should be tested prospectively before operational use. Multimodal fusion should not be assumed to solve the problem automatically; its value will depend on transparency, feasibility, signal quality, ecological validity, and prospective validation.
